# Clinical Evaluation of a Single-Tube Multiple RT-PCR Assay for the Detection of 13 Common Virus Types/Subtypes Associated with Acute Respiratory Infection

**DOI:** 10.1371/journal.pone.0152702

**Published:** 2016-04-04

**Authors:** Dan Zhang, Zhishan Feng, Mengchuan Zhao, Hao Wang, Le Wang, Shuo Yang, Guixia Li, Li Lu, Xuejun Ma

**Affiliations:** 1 Department of Pathophysiology, Guangzhou Medical University, Guangzhou city, Guangdong, China; 2 Key Laboratory for Medical Virology, National Health and Family Planning Commission, National Institute for Viral Disease Control and Prevention, Chinese Center for Disease Control and Prevention, Changping district, Beijing, China; 3 Pediatric Research Institute, Children’s Hospital of Hebei Province, Hebei Medical University, Shijiazhuang, China; 4 Department of Infectious Diseases, Institute of Biomedicine, Sahlgrenska Academy, University of Gothenburg, Gothenburg, Sweden; Kliniken der Stadt Köln gGmbH, GERMANY

## Abstract

Respiratory viruses are among the most important causes of human morbidity and mortality worldwide, especially for infants and young children. In the past years, a few commercial multiplex RT-PCR assays have been used to detect respiratory viruses in spite of the high cost. In the present study, an improved single-tube multiplex reverse transcription PCR assay for simultaneous detection of 13 respiratory viruses was evaluated and compared with a previously reported two-tube assay as the reference method using clinical nasopharyngeal aspirates samples. Of 310 prospectively tested respiratory specimens selected from children hospitalized with acute respiratory illness, 226 (72.90%, 226/310) and 214 (69.03%, 214/310) positive for one or more viruses were identified by the single-tube and the two-tube assays, respectively, with combined test results showing good concordance (Kappa value = 0.874). Individually, the single-tube assay for adenovirus (Adv), human metapneumovirus (HMPV), human rhinovirus (HRV), parainfluenza virus type 1 (PIV1), parainfluenza virus type 3 (PIV3) and parainfluenza virus type 4 (PIV4) showed the significantly superior sensitivities to those of the two-tube assay. No false positives were found. In conclusion, our results demonstrates the one-tube assay revealed significant improvements over the two-tube assay in terms of the better sensitivity, more accurate quality control, less nonspecific amplification, more cost-effective and shorter turn-around time and will be a valuable tool for routine surveillance of respiratory virus infection in China.

## Introduction

Respiratory viruses are among the most common causes of human morbidity and mortality worldwide, especially for infants and young children [1, 2]. The rapid identification is important for both therapeutic and infection control purposes. Diagnoses of viral respiratory tract infection have been performed generally by non-molecular approaches such as direct immunofluorescence and viral culture. They are time-consuming, labor-intensive, and often lack sensitivity or specificity. In the last few years, multiplex RT-PCR assays have been developed to detect respiratory viruses, and many of them have been commercialized, such as xTAG RVP, RVP fast (Luminex Molecular Diagnostics, Toronto, Canada), Resplex II (Qiagen, Mississauga, Canada), FilmArray^®^ Respiratory panel (Idaho Technology Inc., Salt Lake City, UT, USA), Anyplex^™^ II RV16 and Seeplex RV assays (Seegene, seoul, Korea), AdvanSure^™^ real-time RT-PCR (LG Life Science, Seoul, Korea) [3–7]. However, many of these assays are costly, or require specialized laboratory equipment [8, 9].

In our previous study, the two–tube multiplex reverse transcription PCR assay (two-tube assay) to detect sixteen respiratory viruses based on the amplicon size differences using automated electrophoresis system is described. The overall detection rate of the two-tube assay for each virus was comparable to those of the Luminex xTAG RVP Fast assay and Seeplex RV15 ACE assay, demonstrating the high sensitivity and specificity of the two-tube assay in the analysis of clinical samples [10]. However, two-tube assay has a few drawbacks, such as the size difference of some amplicons are not big enough leading to the difficulty in judging the results by untrained staff, the hands-on two-tube assay is till cumbersome and the internal control is not steadily detected, which limits its wide use in the routine screening in provincial and local Center for Diseases Control and Prevention (CDC) in China. In the present study, we adopted the two-tube assay as a reference, and have been progressively optimized and substantially improved the performance of simultaneous detection of thirteen respiratory viruses types/subtypes, the most frequently detected viral agents of respiratory tract infections documented by Beijing Monitoring Network for Pneumonia between 2012–2014 (unpublished data), in a single-tube assay while maintaining excellent sensitivity and specificity. The targeted 13 respiratory viruses types/subtypes including influenza A virus (FluA), influenza B virus (FluB), seasonal influenza A virus subtypes H1N1 2009 pandemic (09H1N1), seasonal influenza A virus subtypes H3N2 (sH3N2), parainfluenza virus type 1 (PIV1), human rhinovirus (HRV), adenovirus (Adv), parainfluenza virus type 2 (PIV2), parainfluenza virus type 3 (PIV3), parainfluenza virus type 4(PIV4), respiratory syncytial virus A (RSVA), respiratory syncytial virus B (RSVB) and human metapneumovirus (HMPV). The aim of this study is to provide a high throughput screening method for routine surveillance of respiratory virus infection in provincial and local Centers for Disease Control and Prevention, China.

## Materials and Methods

### Ethics Statement

All aspects of this study were performed in accordance with national ethics regulations and approved by the Institutional Review Boards of the Center for Disease Control and Prevention of Hebei. Children’s parents were apprised of the study’s purpose and of their right to keep information confidential. Written informed consent was obtained from parents or caregivers.

### Clinical samples

Clinical specimens were obtained from 310 hospitalized patients who had acute respiratory infection(ARI) and admitted to the children's hospital of Hebei, China bet-ween May-October, 2015. Of those 110 (35.4%) were female and 200 (64.6%) were male. Ages were ranged from 1 months to 11 years old, and 279 (90% were under three years old. Trained staff collected nasopharyngeal aspirates (NPA) by adding 3.5ml of transport medium and stored at −80°C.

Total RNA/DNA was extracted from195μL of clinical sample with addition of 5μL of bacteriophage MS2 as an internal control (10^5^ copies) prior to the extraction using the QIAamp Viral RNA Mini Kit (Qiagen, Hilden, Germany). The extracts were eluted into 50μL of DNase- and RNase-free water and stored at −80°C.

### Primers

A total 14 pairs of chimeric primers were added to a single-tube to detect 13 respiratory viruses, and one pair of internal control primer (MS2 F, MS2 R) and one universal primer (Tag) was also added to the tube. The bacteriophage MS2 coat protein derived Virus-like particles (VLPs) was used as an internal control to monitor the extraction and detection process of each specimens [11, 12]. The primers sequences, the target genes, the amplicon sizes, and primer working concentrations are listed in Table 1.

**Table 1:** Primer information.

Primer	Sequence 5–3	Gene	Size(bp)	Concentrations^a^
**FluA F**	**GTACGACTCACTATAGGGA****C ATTGGGATCTTGCACTTGATATT**	**M**	**265**	**100 nM/L**
**FluA R**	**GTACGACTCACTATAGGGA****TTTTTTTTTGTAGAAACAAGGTAGTTTTTTACTC**			
**FluB F**	**GTACGACTCACTATAGGGA****GGGACATGAACAACAAAGATGC**	**NEP/NS1**	**540**	**100 nM/L**
**FluB R**	**GTACGACTCACTATAGGGA****TGTCAGCTATTATGGAGCTG**			
**09H1N1F**	**GTACGACTCACTATAGGGA****GCATTCGCAATGGAAAGAAA**	**HA**	**244**	**100 nM/L**
**09H1N1R**	**GTACGACTCACTATAGGGA****TTTTTTTTCCTCAATCCTGTGGCCAG**			
PIV1 F	GTACGACTCACTATAGGGATCTCATTATTACCYGGACCAA	HA	292	87.5 nM/L
PIV1 R	GTACGACTCACTATAGGGATTTTTTTTCCTGTTGTCGTTGATGTCATA			
PIV2 F	GTACGACTCACTATAGGGATCTACACTGCATCAGCCAGC	HA	195	50 nM/L
PIV2R	GTACGACTCACTATAGGGACCCCTAAAAGAGATGAGCCC			
PIV3 F	GTACGACTCACTATAGGGATTGTCAATTATGATGGYTCAATCT	HA	231	**50 nM/L**
PIV3 R	GTACGACTCACTATAGGGAGACACCCAGTTGTGTTGCAG			
**PIV4 F**	**GTACGACTCACTATAGGGA****CTGAACGGTTGCATTCAGGT**	**Phosphoprotein**	**480**	**50 nM/L**
**PIV4 R**	**GTACGACTCACTATAGGGA****TTGCATCAAGAATGAGTCCT**			
HRV F	GTACGACTCACTATAGGGACCCCTGAATGYGGCTAACCT	5’UTR	145	**100 nM/L**
HRV R	GTACGACTCACTATAGGGACGGACACCCAAAGTAGTYGGT			
HMPV F1^c^	GTACGACTCACTATAGGGACATGCCCACTATAAAAGGTCAG	L	208	100 nM/L
HMPV R1	GTACGACTCACTATAGGGACACCCCAGTCTTTCTTGAAA			
HMPV F2	GTACGACTCACTATAGGGAGAGCTAAYAGAGTGCTAAGTGATG	N	208	50 nM/L
HMPV R2	GTACGACTCACTATAGGGAACTTTCTGCTTTGCTTCCTGT			
Adv F	GTACGACTCACTATAGGGAGCCSCARTGGKCWTACATGCACATC	Hexon	339	100 nM/L
Adv R	GTACGACTCACTATAGGGACAGCACSCCICGRATGTCAAA			
RSVA F	GTACGACTCACTATAGGGACATCCCCTCTATGCACAACC	F	159	50 nM/L
RSVA R	GTACGACTCACTATAGGGACATGTTTCAGCTTGTGGGAA			
RSVB F	GTACGACTCACTATAGGGAAAACGAAGATTTCTGGGCTTC	F	280	100 nM/L
RSVB R	GTACGACTCACTATAGGGATGCGACAGCTCTGTTGATTT			
**H3N2 F**	**GTACGACTCACTATAGGGA****CTATTGGACAATAGTAAAACCGGGRGA**	**HA**	**220**	**50 nM/L**
**H3N2 R**	**GTACGACTCACTATAGGGA****TTTTTTTTTTGTCATTGGGRATGCTTCCATTTGG**			
**MS2 F**	**GTACGACTCACTATAGGGA****GCATGGTTGTCGTCTCTAGGT**	**coat protein**	**351**	**50 nM/L**
**MS2 R**	**GTACGACTCACTATAGGGA****TTTTTTTTTTTTACTTTACGTACGCGCCAGTT**			
**Tag**	**GTACGACTCACTATAGGGA** ^b^			**50 nM/L**

^a^ Primer sequences and primer concentrations varied from two-tube assay are marked in boldface.

^b^ The underlined sequences are universal sequences.

^c^ The primers HMPV-1and HMPV-2 are designed to amplify the L gene and N gene, the amplicon sizes of both PCR products are exactly the same.

### Two-tube Assay and Detection Method

Two-tube assay was performed as described [10]. Briefly, one-step RT-PCR Kit (Qiagen, Hilden, Germany) was used for the amplification. A total of 25μL PCR mixture containing 2μL of extracted RNA and varied primer concentrations was subjected to the following conditions: 50°C for 30min, 95°C for 15min, followed by 10 cycles of 95°C for 30s, 55°C for 30s, and 72°C 30 s; 10 cycles of 95°C for 30s, 65°C for 30s, 72°C for 30s; 25 cycles of 95°C for 30s, 48°C for 30s, and 72°C for 30s, and a final incubation at 72°C for 3min, and the products were analyzed on the QIAxcel automatic electrophoresis using QIAxcel DNA High-Resolution kit.

### The Single-tube Assay and Detection Method

Single-tube assay was performed according to the protocols as described above in two-tube assay with a slight modification of the primer sequences and primer concentration (Table 1).

### Analytical methods

The X^2^-test and Fisher’s exact test were performed to analyze the detection agreement between the single-tube assay and the two-tube assay and resolved discordant results.

## Results

### Performance evaluation using clinical samples

All the comparative detections were double-blind tests performed by trained staff in our laboratory. All of the 310 clinical specimens were simultaneously tested by the single-tube assay and the two-tube assay. The clinical samples are considered as positive when both the targeted pathogen and MS2 are present. The samples are considered to be negative if samples are negative for pathogen but positive for MS2. If both the target and MS2 are absent, the samples are classified as invalid. No invalid sample was found in the optimal single-tube assay. A total of 226 (72.90%, 226/310) and 214 (69.03%, 214/310) samples were detected by the single-tube assay and the two-tube assay, respectively. Viral coinfections were found in 57 specimens (18.38%, 57/310) by the single-tube assay, while in 48 specimens (15.48%, 48/310) by two-tube assay. In our study, PIV3 and HRV were identified to be the viruses most commonly involved in multiple agent infection, while PIV2 and 09H1 were not detected by either of the two methods. All failed results and discordant results were resolved with repeated tests and direct sequencing using the same primers listed in the Table 1.

### Comparison of the assay performance

As shown in Table 2, there were 23 specimens with discordant results between the two-tube assay and the single-tube assay, all of them were confirmed by sequencing as true positives, A total of 24 additional viruses were detected only by the single-tube assay, including 5Adv, 4HMPV, 3HRV, 2PIV1, 9PIV3 and 1PIV4. The two-tube assay does not include the detection of PIV4. The sensitivity, specificity, negative prediction value (NPV), positive prediction value (PPV), the accordance rate, and the kappa value of each virus for the single-tube assay compared to two-tube assay are shown in Table 3.

**Table 2:** The confirmed results for specimens with discordant results between the single-tube assay and the two-tube assay.

Case no.	Two-tube	the single-tube	Confirmed results
10	ND^b^	**PIV3**^a^	PIV3
23	PIV1	PIV1, **HMPV**	PIV1, HMPV
25	ND	**HMPV**	HMPV
26	ND	**HRV**	HRV
29	ND	**Adv**	Adv
68	HRV	HRV, **PIV1**	HRV, PIV1
70	ND	**HRV**	HRV
79	HRV	HRV, **PIV1**	HRV, PIV1
84	HRV,PIV1	HRV, PIV1, **Adv**	HRV, PIV1, Adv
93	HRV	HRV, **PIV3**	HRV, PIV3
99	HMPV	HMPV, **PIV3**	HMPV, PIV3
105	ND	**Adv**	Adv
108	ND	**PIV4**	PIV4
162	PIV3	PIV3, **HRV**	PIV3, HRV
246	HRV	HRV, **Adv**	HRV, Adv
247	HRV,PIV3	HRV, PIV3, **HMPV**	HRV, PIV3, HMPV
252	HRV	HRV, **HMPV**	HRV, HMPV
255	ND	**HRV, HMPV**	HRV, HMPV
279	ND	**PIV3**	PIV3
293	ND	**PIV3**	PIV3
321	ND	**PIV3**	PIV3
378	ND	**PIV3**	PIV3
391	HRV	HRV, **PIV3**	HRV, PIV3

^a^ Discordant results are highlighted in boldface.

^b^ ND stands for not detected.

**Table 3:** Performance of the single-tube assay for individual target compared with the two-tube assay.

	No. of specimens: the single tube assay/ two-tube				Performance of the single tube assay compared with the two-tube assay					
Viruses	+/+	+/−	−/+	−/−	Sensitivity%	Specificity%	PPV%	NPV%	Accordance rate%	Kappa value
FluA	6	0	0	304	100	100	100	100	100	1.00
FLuB	2	0	0	308	100	100	100	100	100	1.00
s09H1N1	0	0	0	310	NA	100	NA	100	100	NA
PIV1	7	2	0	301	100	99.34	77.78	100	99.35	0.87
PIV2	0	0	0	310	NA	100	NA	100	100	NA
PIV3	76	9	0	225	100	96.15	89.41	100	97.1	0.92
PIV4	0	1	0	309	NA	99.68	0	100	99.68	0
HRV	113	3	0	194	100	98.48	97.41	100	99.03	0.98
HMPV	31	4	0	275	100	98.57	88.57	100	98.71	0.93
Adv	11	5	0	294	100	98.33	68.75	100	98.39	0.81
RSVA	9	0	0	301	100	100	100	100	100	1.00
RSVB	9	0	0	301	100	100	100	100	100	1.00
H3N2	0	6	0	304	NA	98.06	0	100	98.06	0

Abbreviation: NA, not applicable.

This table shows the sensitivity, specificity, positive predictive value (PPV), negative predictive value (NPV), and the kappa values for each target using the confirmed results as the reference for comparison. All the accordance rate values were above 97.10%, except for NA, all the kappa values were above 0.75.

## Discussion

Early detection of respiratory virus infections allows clinicians to initiate immediate therapeutic interventions that can reduce complications, antibiotic use, and unnecessary laboratory testing. In this study, we compared the performance of a single-tube multiplex reverse transcription PCR assay with the reference method, a two-tube assay, which has been confirmed to be comparable to the commercial Luminex x TAG RVP Fast assay and Seeplex RV15 ACE detection kit in our previous study. The single-tube assay is also based on the QIAxcel capillary electrophoresis system, which is accessible in most of provincial Centers for Disease Control and Prevention in China. Of the 310 specimens from hospitalized children, a high prevalence of infection and co-infection with the common respiratory viral pathogens were revealed. HRV was found to be most frequently, followed by PIV3. The major discrepancies between the reference two-tube assay and the proposed single-tube assay were the detection rates of HRV, Adv, HMPV, PIV4, PIV1 and PIV3. The 24 viruses detected only by the single-tube assay (5Adv, 4HMPV, 3HRV, and 2PIV1, 9PIV3, 1PIV4) were confirmed by sequencing as true positives. Therefore, the single-tube assay is more sensitive than the two-tube assay for the detection of HRV, HMPV, Adv, PIV, PIV3 and PIV4. The overall detection rate of the single-tube assay for each virus was comparable to that of the two-tube assay (kappa>0.75) demonstrating the high sensitivity and specificity of the single-tube assay in the analysis of clinical samples.

Compared with the two-tube assay, the single-tube assay revealed better sensitivity, more accurate quality control, less nonspecific amplification, more cost-effective and shorter turn-around time. This improvement might be attributed to several aspects as follows. First, we have replaced human genome RNase P gene in the two-tube assay with bacteriophage MS2 as an internal control. The utility of MS2-based armored RNA as an assay internal control has been documented in many clinical applications [13, 14]. In this study, the internal control MS2 was added to each specimen prior to extraction as an exogenous control to monitor the whole process from nucleic acid extraction to RT-PCR. Second, we inserted a few T nucleotides when appropriate between the specific and universal sequences in the primer design to ensure each amplicon can be easily distinguished by the QIAxcel machine. Third, homologous tail sequences was added to the 5' end of each chimeric primer [15]. The homo-tag assisted non-dimer (HAND) system introduced in this study was to prevent dimer formation and allow more, specific, sensitive, and stable detection of the viruses [16]. Fourth, for detection of multiple viral targets, simultaneous presence of multiple targets in one specimen may have led to competitive inhibition of amplification of less abundant targets and may explain some loss of assay sensitivity. In this study, we avoided this problem by optimizing temperature switch PCR parameters and further fine-tuning the concentration of primers. In addition, single-tube assay is more convenient and rapidly to apply to clinical specimens, saving the cost and turn-around time.

In our study, the most frequently detected pathogen was HRV, while in other study, the RSV was the most commonly detected viral pathogen [17, 18]. This difference might be caused by the following two aspects. First, the detection of RSV increased during the winter, whereas HRV was detected year-round [19, 20]. In this study, the samples collection time is not in winter (May to October). Second, HRV is a well-recognized cause of common colds and associated with upper respiratory tract complications. In this study, the patients were hospitalized for both the upper respiratory tract and lower respiratory tract infections.

The two-tube assay does not include PIV4 while single-tube assay does. For PIV4 and PIV2, only one PIV4 specimen was detected as positive and no PIV2 were detected by the single-tube assay with acute respiratory illnesses, which is consistent with previous reports that PIV2 and PIV4 were not identified as frequently as PIV1 and PIV 3 [21–23]. The different geographic location might also lead to the different seasonal distributions of PIVs. All of the FluA positives detected by the single-tube assay were subsequently sequenced to be H3N2 infection. 09H1N1 were not detected by either of two methods because H3N2 is the predominant seasonal FluA circulating in 2015 in China. Though PIV2 and 09H1N1 were not found in the samples, we detected a few stock clinical samples previously identified as 09 H1N1 or PIV2 using both single-tube and two-tube assays, and the results demonstrated both assays worked very well (data not shown). Moreover, no false positives were found by the single-tube assay, further suggesting the high specificity of the single-tube assay.

However, the limitations of our study are that only the main causes of respiratory infection were investigated. Other respiratory tract infection related viruses, such as coronaviruses 229E, HKU1, NL63, and OC43, human bocavirus (HBoV), enteroviruses (EV) [17, 24, 25], are not included in this study. Besides, the association of clinical outcome with the etiology information, particularly the role of co-infections in the clinical course and severity is not addressed. Given the high prevalence (18.38%, 57/310) and diversity of viral co-infections in our study, further research is also needed.

In summary, the improved single-tube assay in this study using automatic capillary electrophoresis is a rapid, cost-effective and reliable method with high sensitivity and specificity for the simultaneous detection of thirteen respiratory virus infections, and will be a valuable tool for routine surveillance of respiratory virus infection in China.
